# Gut remediation: a potential approach to reducing chromium accumulation using *Lactobacillus plantarum* TW1-1

**DOI:** 10.1038/s41598-017-15216-9

**Published:** 2017-11-08

**Authors:** Gaofeng Wu, Xingpeng Xiao, Pengya Feng, Fuquan Xie, Zhengsheng Yu, Wenzhen Yuan, Pu Liu, Xiangkai Li

**Affiliations:** 1 Ministry of Education Key Laboratory of Cell Activities and Stress Adaptations, School of Life Science, Lanzhou University, Tianshuinanlu #222, Lanzhou, Gansu 730000 People’s Republic of China; 2 Department of Development Biology Sciences, School of Life Science, Lanzhou University, Tianshuinanlu #222, Lanzhou, Gansu 730000 People’s Republic of China; 3 Department of Oncology Surgery, First Hospital of Lanzhou University, Lanzhou, Gansu 730000 People’s Republic of China

## Abstract

Some lactobacilli have protective effects against some heavy metals in mammals, but the underlying mechanism is not fully understood. To evaluate the remediation potency and the mechanism of Lactobacillus against chromium (Cr) in mice, *Lactobacillus plantarum* TW1-1 was orally administrated to Kunming mice for 7 weeks during exposure to 1 mM K_2_Cr_2_O_7_ in drinking water. Results showed that TW1-1 helped to decrease Cr accumulation in tissues and increase Cr excretion in feces, and may also attenuate alterations in oxidative stress and histopathological changes caused by Cr exposure. Moreover, the chromate reduction ability of fecal bacteria doubled after administration of TW1-1 upon Cr induction. MiSeq sequencing of fecal bacterial 16S rRNA genes revealed that the overall structures of gut microbiota was shifted by Cr exposure and partially restored by TW1-1. The abundances of 49 of the 79 operational taxonomic units altered by Cr were reversed by TW1-1. Based on these, we proposed a working model of TW1-1 against Cr: TW1-1 helps to remove Cr from the host and meanwhile acts as a regulator of gut microbiota, which aids in chromate reduction and provide protection against Cr. We call this process of remediation of heavy metal in the gut “gut remediation”.

## Introduction

Contamination of agricultural products by heavy metals is a serious problem globally. Most heavy metals in the greater environment derive from mining activities; irrigation; solid-waste disposal; pesticides and fertilizers; and atmospheric deposition^1^. Heavy metals in the soil are difficult to remove with current remediation methods. Crops growing in soils contaminated with metals will subsequently be contaminated as well, and their consumption by humans may result in a suite of health problems that range in severity^2^. It has been reported that there are up to 2.5 million potentially contaminated sites in Europe alone^3^; for example, 700 km^2^ of the Campine region of Belgium and the Netherlands have been diffusely contaminated by atmospheric deposition of cadmium (Cd), zinc, and lead (Pb)^4^. In China, a total of 2.88 × 10^6^ ha of land has been contaminated with heavy metals as a result of mining, with an additional mean area of 46,700 ha polluted annually^5^. A classic example of the health effects of consumption of crops grown in areas polluted with heavy metals is the ‘itai-itai’ disease in Japan, which was traced to the consumption of rice and soybean grown in soil heavily polluted with Cd^6^. Such examples highlight the extent of the challenge and the importance of mitigating heavy metal pollution of crops.

A number of countermeasures for remediation of heavy metal contaminated soils have been introduced over the past several decades, including physical, chemical, and microbial techniques, and phytoremediation. Removal of heavy metals using living organisms is a particularly useful approach^7^. Microbial remediation has certain advantages, such as greater public acceptance, lower costs, and minimal site disruption^8,9^. Phytoremediation is another effective technique^10^, as it eliminates the need for soil excavation and transport^11^. However, the total area remediated by phytoremediation and microbial remediation is far smaller than the total area of contamination, and thus alternative approaches for the mitigation of heavy metal pollution are urgently needed.

Human exposure to heavy metals is currently inevitable. Some lactic acid bacteria such as lactobacilli are known capable of binding and removing heavy metals *in vitro* ^12^. As such, heavy metal remediation via supplementation with lactobacillus has been studied. Previous researches have found that oral administration of *Lactobacillus plantarum* (*L. plantarum*) can protect mice against toxicity of Cd, Pb, copper, and aluminum by facilitating the excretion of heavy metals through feces and inhibiting absorption via intestinal barrier^13–16^. Further references on how *L. plantarum* remediate heavy metals *in vivo* are scarce. On the other hand, a previous study using germ-free mice has shown that intestinal microbiome plays an essential role in fending off heavy metals exposure^17^. Ingestion of Cd, Pb, and nickel can lead to alterations in the composition of gut microbiota^18–20^. Hence, whether oral supplementation with *L. plantarum* could protect against heavy metal toxicity by regulating the composition and function of intestinal microbiota deserves to be investigated.

Chromium (Cr) is a common toxic heavy metal that is both mutagenic and carcinogenic to humans^21^. In the present study, we examined the protective effects of *L. plantarum* TW1-1, a candidate probiotic strain derived from fermented dairy products with known Cr reduction capability, against Cr toxicity in mice, and demonstrated that TW1-1 can effectively attenuate Cr(VI) toxicity^22^. Furthermore, we evaluated the effectiveness of “gut remediation", a process that involves both direct and indirect remediation of heavy metal pollution by *L. plantarum*.

## Results

### Cr(VI)-reduction ability of Lactobacillus strains

**Figure 1:**
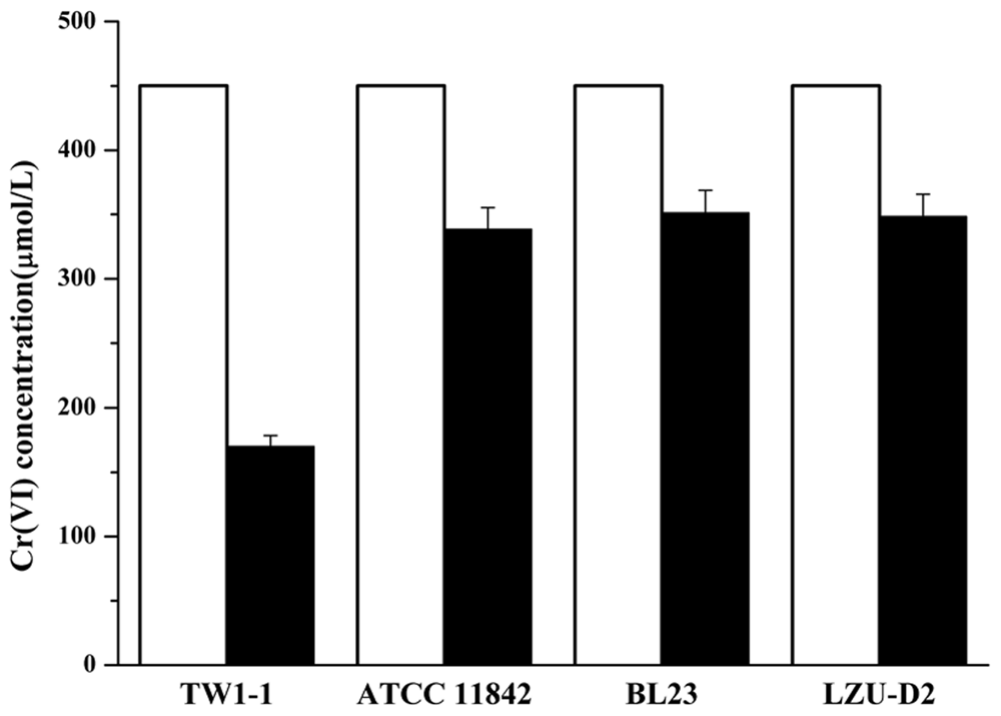
Cr(VI) reduction abilities of four strains. (□) Initial Cr(VI) concentration; (■) Cr(VI) concentration after incubation for 48 h.

Four lactobacillus strains capable of reducing Cr(VI) were identified from, including *L. plantarum* TW1-1, *L. delbrueckii* ATCC 11842, *L. casei* BL23, and *L. paracasei* LZU-D2. Phylogenetic analysis of these four strains was performed using the neighbor-joining method (Fig. S1). The Cr(VI)-reduction abilities of the four strains are shown in Fig. 1. Strain TW1-1 reduced 60% of 0.5 mM Cr(VI) within 48 h of incubation, whereas strains BL23, ATCC11842, and LZU-D2 reduced about 20% of Cr(VI) over the same time period. The precipitates were re-oxidized by Na_2_S_2_O_3_, with results indicating that Cr(VI) was reduced rather than absorbed (Fig. S2). Thus, strain TW1-1 was chosen for further experimental study in mice, and strain LZU-D2 was used as a control lactobacillus strain with less Cr-reducing ability.

### Cr levels declined in tissues and increased in feces following TW1-1 treatment

**Figure 2:**
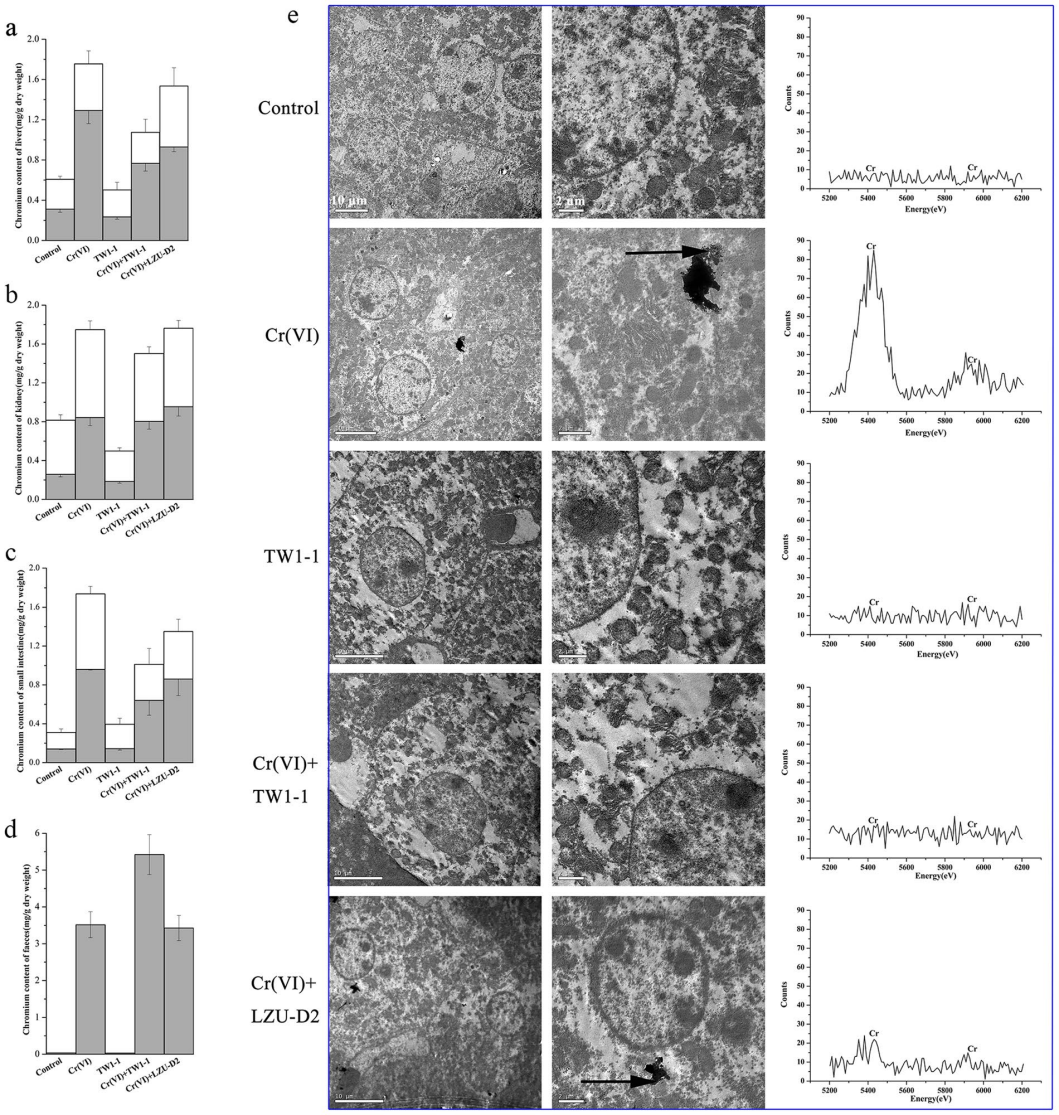
(**a**) to (**d**) Determination of Cr content in tissues and feces by atomic absorption spectrophotometer. (□) Cr(VI) content in tissues and feces; () Cr(III) content in tissues and feces. Cr(III) content was detected via sodium hydroxide precipitation. (**e**) TEM images of hepatic tissue of mice and EDX data analysis. Arrows show accumulations of Cr particles. The particles are located on the cell membrane in Cr(VI) group, and in the cytoplasmic matrix and near the cell nucleus in Cr(VI) + LZU-D2 mice. Scale bars (from left to right in each row) are 10 μm and 2 μm. All images were taken at 150 kV.

The growth of mice from all five groups were monitored throughout the experiment and did not exhibit significant differences between groups (Fig. S3). Cr (Cr[VI] + Cr[III]) content in tissues and feces were measured after mice were sacrificed. After Cr(VI) treatment, Cr levels were found sharply increased (Fig. 2a–d). Concentrations of Cr(VI) in the kidneys and liver were twice that of control mice, and concentration in the small intestine increased from 0.1405 mg/g to 0.9602 mg/g. Concentrations of Cr(III) in the kidneys and liver were four-fold higher than in mice in the control group, and concentrations in the small intestine rose from 0.1703 mg/g to 0.7774 mg/g. However, both Cr(VI) and Cr(III) levels were lower in the liver, kidneys, and small intestines of mice treated with Cr(VI) + TW1-1 than in mice treated solely with Cr(VI). At the same time, Cr(III) content was markedly increased in feces with TW1-1 treatment, rising from 0.0325 mg/g to 3.5166 mg/g, whereas Cr(VI) was nearly absent (Fig. 2d). In addition, administration of LZU-D2 also resulted in reductions in Cr concentrations in tissue samples, albeit to a lesser degree. The Cr found in feces was in the form of Cr(III), suggesting that most of the Cr(VI) entering the body via oral intake was reduced to Cr(III) and then directly excreted in the feces. Therefore, it would appear that TW1-1 is capable of reducing Cr concentrations in body tissues.

Rates of Cr accumulation in the hepatic cells were further shown by transmission electron microscopy (TEM) images in Fig. 2e. Dark-colored precipitates were observed on the cell membrane of hepatocytes of mice treated with Cr(VI), and lower levels of Cr accumulation were revealed in the Cr(VI) + TW1-1 and Cr(VI) + LZU-D2 groups, although the effect was more apparent in the former group.

### Strain TW1-1 attenuated Cr(VI)-induced oxidative stress and inflammation

**Figure 3:**
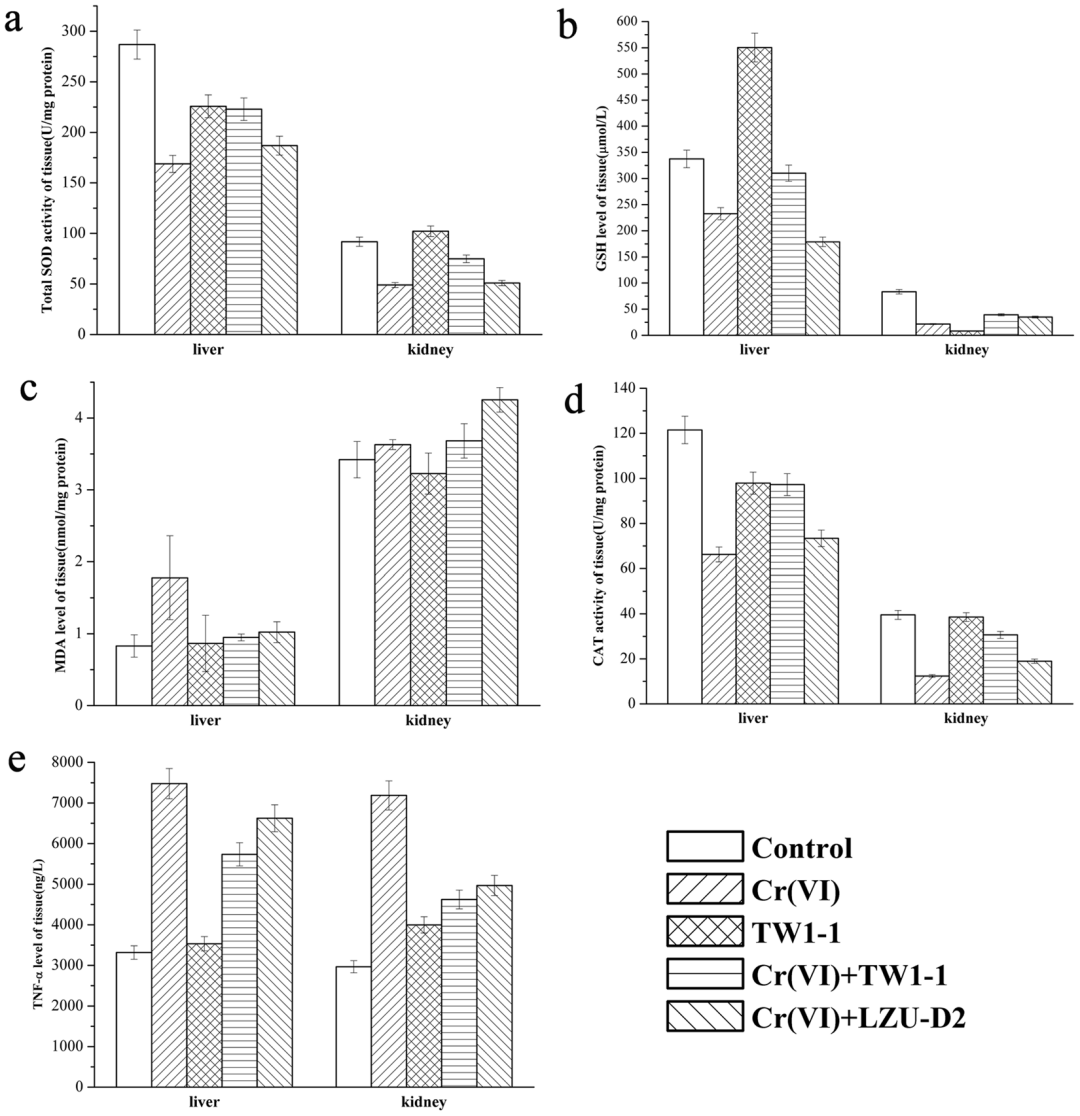
Effects of TW1-1 on Cr-induced alterations of the activities of some biomarkers in livers and kidneys of mice.

The levels of total superoxide dismutase (T-SOD), glutathione (GSH), malonaldehyde (MDA), catalase (CAT), glutathione disulfide (GSSG), and tumor necrosis factor α (TNF-α) were assayed in mouse tissues (Fig. 3 and Fig. S4). In the liver, MDA levels increased in mice treated solely with Cr(VI), and levels of GSH, CAT, and T-SOD decreased. With TW1-1 treatment, however, the levels of these markers were greatly restored (P < 0.01). In the kidney, similar restorative effects of TW1-1 were observed. Cr(VI) exposure also resulted in a marked increase of the inflammatory cytokine TNF-α in various tissues (P < 0.01), and TW1-1 reversed the changes in TNF-α to a great degree, especially in the kidneys (P < 0.05) (Fig. 3e). Considered together, our results suggested that TW1-1 was effective in mitigating oxidative stress and inflammation caused by exposure to Cr(VI).

### Tissue damages induced by Cr(VI) were effectively alleviated after TW1-1 intervention

**Figure 4:**
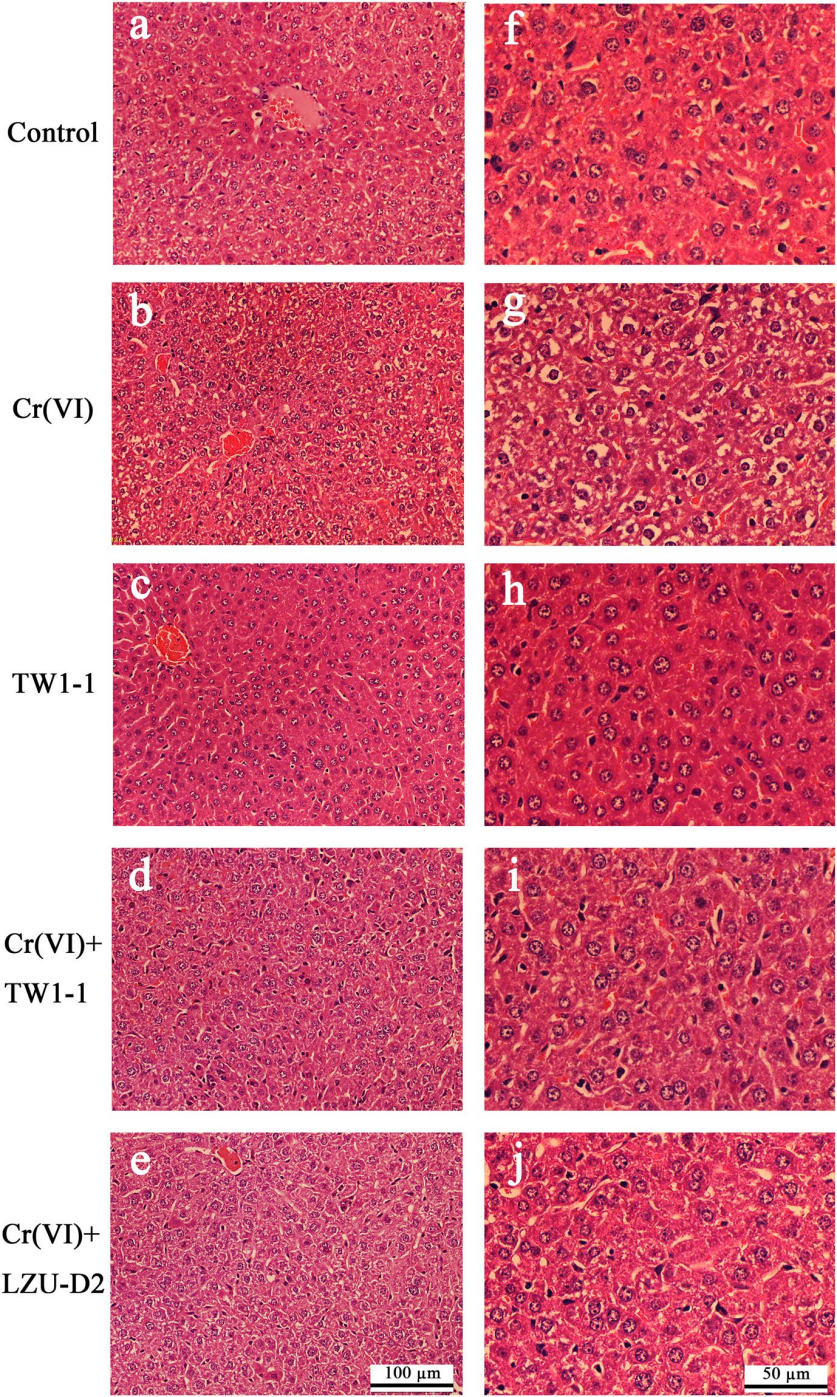
Representative photomicrographs of H&E staining of mouse liver tissues. (**a**),(**f**): Normal appearance of hepatic tissue in the control group. (**b**),(**g**): Clear Cr-induced alterations with loss of intact liver plates, hepatocyte vacuolization, and chromatin condensation. (**c**),(**h**): TW1-1 only group with no apparent histological alterations. (**d**),(**i**): Hepatocyte vacuolization and chromatin condensation were greatly alleviated in Cr(□) + TW1-1 group. (**e**),(**j**): Moderate histological restoration in Cr(VI) + LZU-D2 group. Magnification in panels (**a**) to (e**)** is 12.6×, and magnification in (**f**) to (**j**) is 25.2×. Scale bar in **e** represents 100 μm; scale bar in**j** represents 50 μm.

Hepatic tissues collected from mice in the control group appeared to be normal, whereas those of mice administered Cr exhibited a variety of pathologic alterations, including loss of intact liver plates, hepatocyte vacuolization, and chromatin condensation in the liver (Fig. 4). Co-treatment with TW1-1 resulted in restoration of a close-to-normal appearance of liver tissues. And moderate histological restorations were observed in mice treated with LZU-D2. There were no obvious histological alterations detected in the TW1-1-only group. Taken together, these results indicated that TW1-1 could be used to alleviate Cr (VI)-induced hepatic damages safely and effectively.

### Colonization of TW1-1 increased the Cr(VI)-reduction ability of fecal microbes

**Figure 5:**
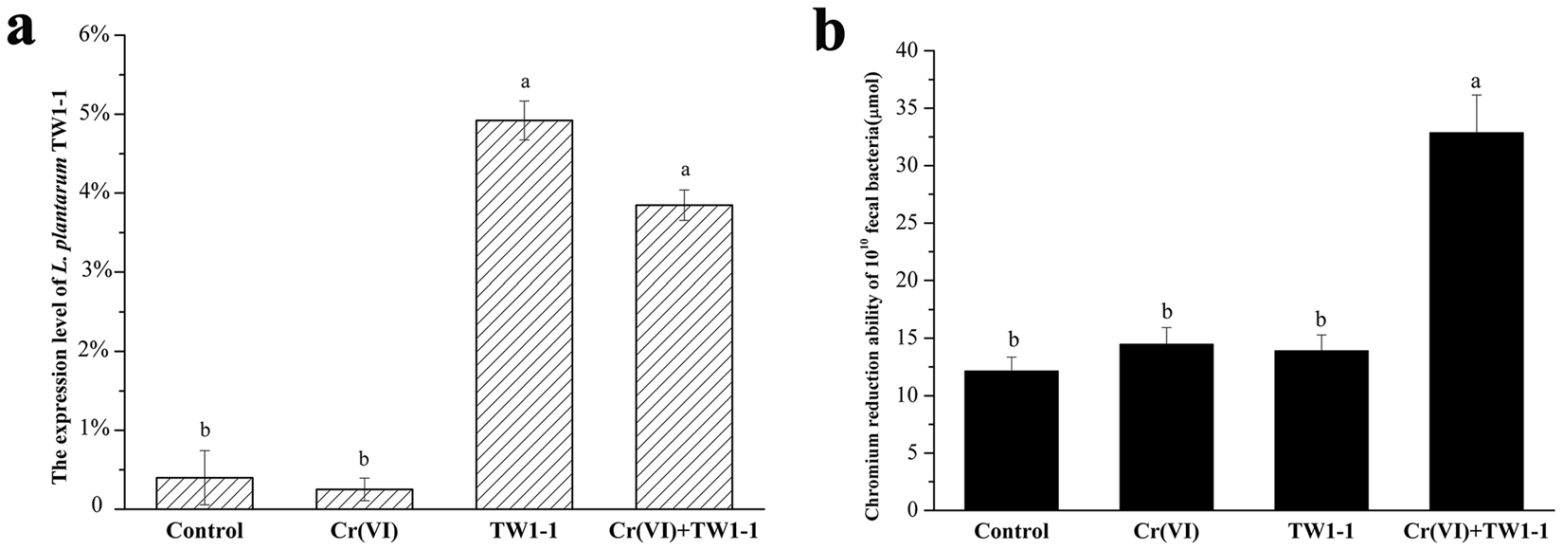
Quantification of TW1-1 in culturable bacteria of feces inoculated in MRS medium (**a**) and Cr reduction ability of feces of four groups *in vitro* at the same time (**b**). The bars in **b** represent the reduction ability of 10^10^ fecal bacteria. Same letters indicate no significant differences (*P* > *0.05*); different letters indicate a significant difference (*P* < *0.05*).

One important trait of probiotics is their ability to survive transit through the upper gastrointestinal tract. As such, we investigated levels of TW1-1 in feces using real time (RT)-quantitative (q) PCR. The relative expression level (the expression level of TW1-1/the expression level of total bacteria) of TW1-1 was significantly higher in mice administered TW1-1 (P < 0.01) (Fig. 5a), indicating that TW1-1 colonized in the gastrointestinal tracts of treated mice.

To further investigate whether TW1-1 enhanced the reduction of Cr(VI) of gut microbiota, we compared the Cr(VI)**-**reduction ability of fecal microbes between the groups. As shown in Fig. 5b, the ability of fecal bacteria to reduce Cr(VI) doubled in mice given Cr diets supplemented with TW1-1 (P < 0.01), an indication that TW1-1 indeed enhances the ability of gut microbiota to reduce Cr(VI). Interestingly, the enhancement was only observed in Cr(VI) + TW1-1 group but not in the TW1-1-only group.

### Structural changes of the gut microbiota in response to Cr(VI) treatment and probiotic intervention

**Figure 6:**
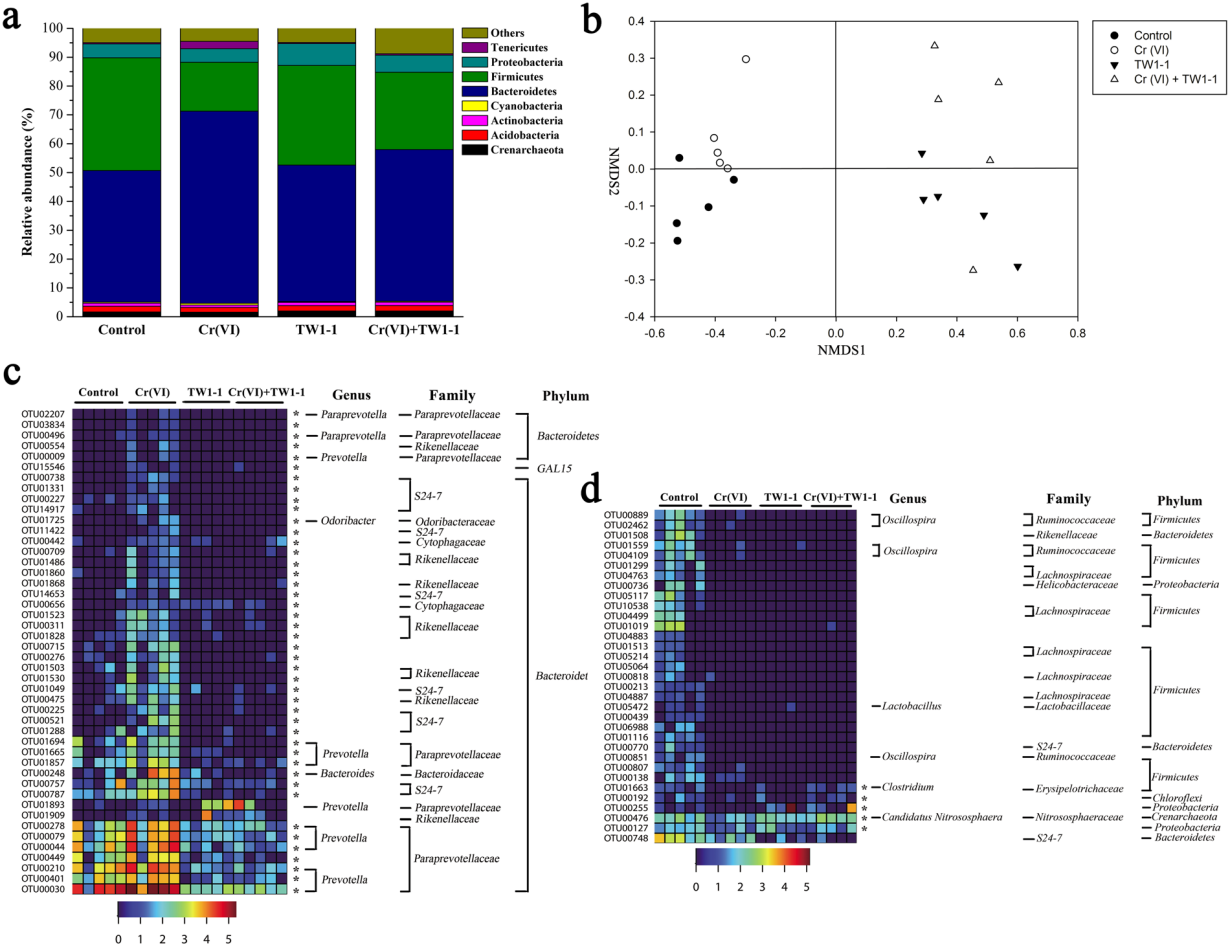
Analysis of MiSeq data by R. (**a**) Relative abundance (% of total reads) of bacterial 16S rRNA gene at phylum level. (**b**) NMDS analysis of OTUs. (**c**) and (**d**) 79 OTUs that were changed in abundance by Cr exposure according to redundancy analysis. Heatmap of the abundance of 46 OTUs enriched and 33 OTUs reduced by Cr(VI). The taxonomy of the OTUs (genus, family, and phylum) is depicted on the right. *OTUs in which abundance was changed by Cr(VI) and then reversed by TW1-1.

20 fecal samples were collected after a 7-week treatment and subjected to MiSeq sequencing of the bacterial 16 S rRNA gene V4 region, with 4,794 reads generated from each sample. The majority of the phyla consisted of *Bacteroidetes*, *Firmicutes*, *Acidobacteria*, and *Proteobacteria* in all groups (Fig. 6a). Seven weeks of Cr(VI) treatment induced significant changes in gut microbial community structure, with the relative abundances of *Bacteroidetes* (45.62% to 66.64%, P = 0.0015) and *Tenericutes* (from 0.52% to 2.54%, P = 0.0285) increasing, and the abundance of *Firmicutes* significantly declined (39.07% to 16.97%, P = 0.0006). Cr(VI) supplemented with TW1-1, however, partially reversed the effects of Cr(VI) on the microbial abundance at phylum level, with decreased abundance of *Bacteroidetes* (66.64% to 52.63%, P = 0.0315) and increased abundance of *Firmicutes* (16.97% to 26.8%).

An NMDS was then performed to examine the relationships among the four groups. As shown in Fig. 6b, samples segregated into four distinct groups based on treatment type, suggesting that treatment had an influence on the composition of the bacterial community; the microbial communities of the two TW1-1 groups were determined to be more closely related to one another than to the control and Cr(VI)-only groups.

Treatment with Cr(VI) significantly altered the abundance of 79 OTUs, as compared to that in the control group, with 46 OTUs enhanced and 33 OTUs reduced (Fig. 6c–d). Most of the OTUs assigned to *Bacteroidetes* were enriched as a result of Cr(VI) treatment, whereas the majority of OTUs that decreased were associated with *Firmicutes*. Administration of TW1-1 partially reversed the abundances of 49 of the 79 OTUs altered by Cr(VI) treatment.

### Modulation effects of TW1-1 intervention on gut microbiota

Sequences data were analyzed at the family, genus and species levels as well (Tables S2 and S3, Fig. S5). At the family level, Cr exposure led to increases in the proportion of *Paraprevotellaceae* (from 3.76% to 9.04%, P = 0.036) and S24-7 (from 16.38% to 25.09%), whereas the relative abundance of *Lachnospiraceae* declined (from 6.34% to 2.22%, P = 0.007). Co-treatment with TW1-1 significantly reversed the effects of Cr(VI) on the microbial abundance, with decreased abundance of *Paraprevotellaceae* (9.04% to 1.72%, P = 0.012) and increased *Lachnospiraceae* (2.22% to 6.51%, P = 0.00009) at the family level. Overall, TW1-1 mitigated many of the effects that Cr(VI) treatment had on microbial populations, with the exception of the genus *Lactobacillus*.

Supplementation with TW1-1 alone led to alterations in the proportion of *Proteobacteria* at the phylum level, and increased relative abundance of the *Bacteroidaceae*, *Prevotellaceae*, S24-7 and *Paraprevotellaceae* (from 3.76% to 9.04%, P = 0.036) at the family level, and changes in the proportion of genera *Prevotella* and *Faecalibacterium*. In general, both Cr(VI) and TW1-1 treatment respectively led to changes in the relative abundance of some bacterial species.

## Discussion

In this study, we investigated the effects and the mechanisms of a Cr-reducing strain TW1-1 in remediating heavy metal Cr in mouse.

Some lactobacilli are Cr-resistant and would be useful for Cr detoxification and bioremediation, e.g. *L. paracase* CL1107 manifested *in vitro* Cr(VI) reduction ability, though not as competent as TW1-1^23,24^. TW1-1 reduced tissue absorption of Cr by promoting its excretion through feces, implying that TW1-1 might exert its effects in two possible ways. First, inhabiting TW1-1 might quickly reduce Cr(VI) entering through the digestive tract to the less soluble Cr(III), thus reducing the amount of Cr absorbed via the intestinal tract and increasing the amount of Cr excreted with feces. This protective mechanism of TW1-1 against Cr accumulation in mice is similar to several reports focusing on *L. plantarum* strains^25,26^. Cr(VI) toxicity can be attributed to its high solubility, rapid permeability through cell membranes, and subsequent interaction with intracellular proteins and nucleic acids^27^, whereas Cr(III) is much less soluble and less able to permeate through cell membranes; as such, reducing Cr(VI) to Cr(III) would “trap” Cr in microbes and ultimately it would be expelled from the body along with these microbes. Second, live TW1-1 might improve bowel movement activity, which is suppressed by Cr, thus increasing fecal excretion of Cr^25^.

The effects of Cr(VI) exposure on oxidative stress and inflammation was evidenced by the decrease in the levels of such antioxidant markers as T-SOD, CAT, and GSH, and the increase in the peroxidation marker MDA, in body tissues. These results corroborate those of previous research in rats^28^. MDA is an end product and marker of the lipid peroxidation process, whereas SOD and GSH are thought to be important components of the host’s antioxidant defense system^29^. The exhaustion of SOD enzymes might be due to the sharp increase in ROS caused by Cr exposure, which may exceed the anti-oxidative capacities of both SOD and CAT^30^. Increasing levels of TNF-α suggested that host tissues might undergo further damage in response to Cr-induced stress^31^. Our work demonstrated that TW1-1 could partially reverse the changes in the biomarkers induced by Cr(VI) exposure.

To gain some insight on the mechanism of TW1-1-aided remediation, Cr(VI)-reduction ability of fecal microbes of Cr(VI) + TW1-1 mice was measured and found enhanced. Specifically, the capacity of 10^10^ fecal bacterial cells in Cr(VI) + TW1-1 mice to reduce Cr(VI) was approximately 18 μM higher than in mice treated solely with Cr(VI) (P < 0.01), which may be attributed to the Cr(VI)-reducing capacity of TW1-1 cells in the fecal bacteria or to functional changes of the gut microbiota as a whole. Based on the reducing ability of TW1-1 strain as shown in Fig. 1 (roughly 3 μM Cr(VI)/10^10^ TW1-1 cells after 48 h), we estimated that the 18 μM increase in the reducing ability of fecal bacteria in Cr(VI) + TW1-1 group was unlikely solely due to the reducing capacity of TW1-1; more likely, TW1-1 intake causes structural and functional changes in the gut microbiota, which in turn enhance rates of Cr(VI) reduction. The profiling changes of RNA and proteins in the gut microbial community in response to TW1-1 intervention deserve further investigation. In addition, enhanced of Cr(VI) reduction was not observed in group supplied with TW1-1 alone, suggesting that the intake of Cr(VI) was necessary for the induction of Cr resistance in the fecal microbes, consistent with a previous study showing that heavy metal remediation genes are upregulated by Cr(VI) treatment in soil microbiota^32^.

Given the phenotypic results, gut microbial populations were analyzed to further explore the protective mechanisms of candidate probiotic TW1-1. Gut microbiota is characterized by temporal stability and resilience, however many environmental perturbations that exceed the resilience capacity of gut microbiota can lead to dysbiosis^33^. In our case, mice were treated with 100 mg Cr/L water, a dosage that mimics acute Cr(VI) intoxication in human. The results showed that high doses of Cr(VI) led to dysbiosis of the gut microbiota, with significant shifts in the relative abundances of the *Bacteroidetes* and *Firmicutes*; similar transformations in microbial community composition have been observed in mice treated with Cd(II) and Pb(II)^34^. Moreover, Cr(VI) treatment increased the abundance of S24-7^35^, *Prevotella*, and *Clostridiales*, but lowered *Lachnospiraceae* abundance. It was previously reported that *Prevotella* are involved in the transferable and colitogenic activity in a colitis mouse model^36,37^. *Clostridiales* are involved in hepatic metabolic activity and immune function, and affect the production of short chain fatty acids^38,39^. The *Lachnospiraceae* produce butyrate, which plays an important role in energy supply and the development of intestinal epithelial cells; lower abundances might thus correlate with the inflammation and oxidative stress observed in mice exposed to Cr(VI)^40,41^. Supplementation with TW1-1 restored the abundance of *Bacteroidetes* and *Firmicutes* to almost normal levels, decreased concentrations of both unclassified S24-7 and *Prevotella*, and increased the abundance of unclassified *Lachnospiraceae* to weaken the adverse effects of Cr(VI) exposure. Overall, our results suggested that TW1-1 could partially repair gut microbiota dysbiosis caused by Cr(VI) possibly by enriching the abundance of species beneficial to health and suppressing harmful species.

Ingestion of TW1-1 resulted in major structural changes of the gut microbiota, but did not lead to dysbiosis of gut microbiota in mice, a finding consistent with those previously reported^42^. Administration of TW1-1 led to increases in the abundance of *Proteobacteria*, which are involved in the metabolism of carbohydrates and maintenance of normal physiological functioning^43^, and the abundance of *Faecalibacterium*, which presumably play a protective role in the intestine^44^. Other species known for their anti-inflammatory effects increased in response to TW1-1 as well, including *Faecalibacterium prausnitzii* ^45^ and *Prevotella copri* ^46^. Generally, TW1-1 administration did not trigger inflammation, oxidative stress, or obvious dysbiosis of gut microbiota, suggesting that TW1-1 could be a potential candidate in remediating heavy metals with no obvious harmful side effects.

**Figure 7:**
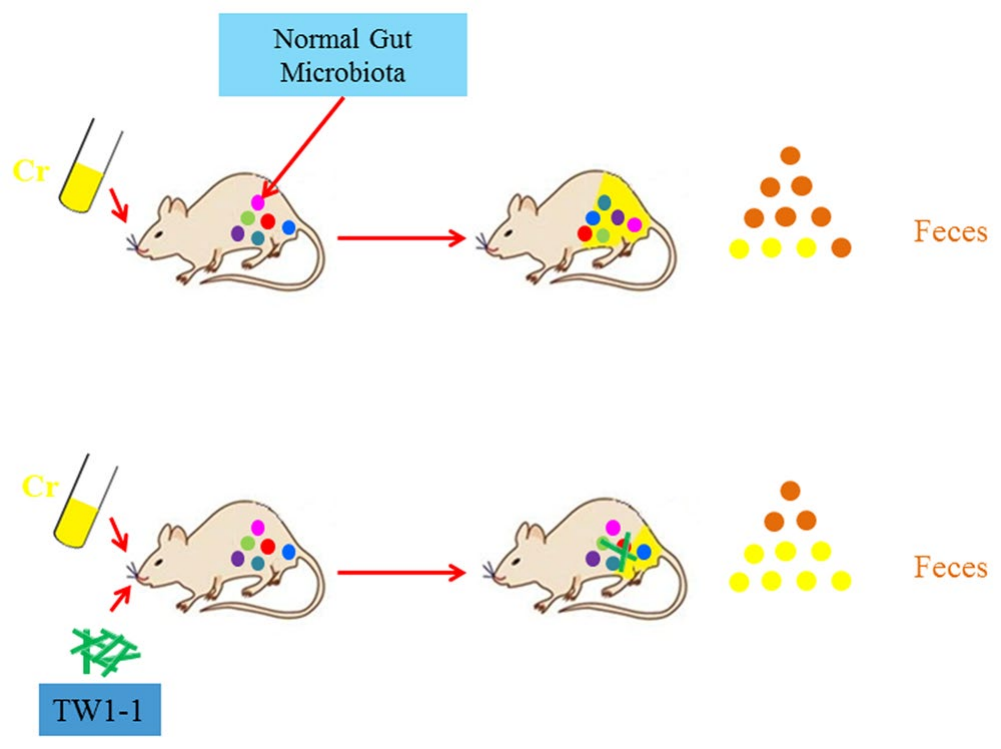
A proposed working model of gut remediation of Cr. Intake of TW1-1 facilitated Cr excretion and helped to maintain the homeostasis of gut microbiota.

In conclusion, our study demonstrated the utility of *L. plantarum* TW1-1 for attenuating Cr(VI)-induced toxicity in mice. TW1-1 might remediate heavy metal toxicity by modifying the structure and functioning of the gut microbial community, a process we termed “gut remediation” (Fig. 7). Compared to current remediation technology, gut remediation appears to be direct and efficient at repairing tissue damages caused by heavy metal pollution, and might play a role in treating heavy metal toxicity in human.

## Methods

### Bacteria and media

The strains *L. delbrueckii* ATCC 11842^47^, *L. casei* BL23^48^, and *L. plantarum* TW1-1^22^, originally derived fermented dairy producted, were provided by Dr. J. Kong (Shandong University, Jinan, China), and *L. paracasei* LZU-D2^49^, isolated from fermented milk of yak from Tibetan plateau, was obtained from Dr. X. Guo (Lanzhou University, Lanzhou, China) (Table 1). The Genebank ID of 16S rRNA gene sequence of TW1-1 is KJ026561.1. A de Man, Rogosa, and Sharpe (MRS, Beijing Solarbio Science & Techinology, Beijing, China) growth medium was used to incubate the bacteria. A solid medium was prepared by adding 2% (w/w) agar to the MRS medium. Stock solutions of potassium dichromate (Shuangshuang Chemical Reagent Company, Yantai, China) were prepared at 1 mM in distilled water and 2 mM in MRS medium.

**Table 1:** Strains. NCBI database accession number (http://www.ncbi.nlm.nih.gov/).

Strains	Organism	Genus	Family	Accession Number
TW1-1	*Lactobacillus plantarum* subsp. plantarum	*Lactobacillus*	*Lactobacillaceae*	KJ026561
LZU-D2	*Lactobacillus paracasei*	*Lactobacillus*	*Lactobacillaceae*	KX814367
BL23	*Lactobacillus casei*	*Lactobacillus*	*Lactobacillaceae*	AF385770
ATCC 11842	*Lactobacillus delbrueckii* subsp. Bulgaricus	*Lactobacillus*	*Lactobacillaceae*	NR_115131

### Determination of Cr(VI)-reducing abilities of Lactobacillus strains and fecal microbes *in vitro*

All strains were cultivated in MRS at 37 °C for 24 h under aerobic conditions, following which cultures were centrifuged and washed before being re-suspended in 0.9% NaCl solution. Re-suspended cells were inoculated in saline solution containing 500 µmol/L Cr(VI). After 48 h, cultured cells were centrifuged, with concentrations of Cr(VI) in the supernatant determined using the DPC technique^50^.

To determine Cr(VI) reduction in feces, approximately 0.1 g of feces pellets were collected from two mice in each group, and re-suspended in 5 ml of MRS medium and incubated overnight at 37 °C in anaerobic chamber. Next, 1 ml of the culture were inoculated in 100 ml of MRS medium containing 100 µM of Cr(VI) and incubated for additional 12 h at 37 °C under anaerobic conditions. The solution was then centrifuged to obtain cells and washed with PBS. Cells were then cultured in 100 ml of PBS containing 500 µM Cr(VI) anaerobically. After 48 h, samples were centrifuged; the supernatant was used to determine Cr(VI) concentrations, and the cells were used to obtain DNA for use in the qRT-PCR. The absolute numbers of total bacteria in each feces sample were calculated based on qRT-PCR results.

### Animals, experimental design

Fifty adult female Kunming mice with an average weight of 26 g were purchased from the Animal Facility of the Medical School of Lanzhou University (Lanzhou, China), and maintained in a 12 h light/dark cycle. After 1 week of acclimatization and diet adaptation, the mice were randomly divided into five groups, with 10 mice per group (Table 2). Group 1 served as the control (no exposure to Cr[VI] or TW1-1), and Groups 2, 3, and 4 were administered 1 mM K_2_Cr_2_O_7_ in drinking water based on our pilot study. In addition to Cr(VI), mice in Groups 3 and 4 were administered TW1-1 and LZU-D2 via oral gavage, respectively, at the rate of 1 × 10^9^ CFU/mouse/every second day for 7 weeks. Mice in Group 5 were administered TW1-1 only. Bacterial suspensions were prepared as previously described^51^. Solutions of Cr(VI) and water were replaced twice weekly with fresh preparations. All procedures and protocols followed in this study conform to the institutional guidelines and were approved by the Ethics Committee of Lanzhou University.

**Table 2:** Experimental animal protocol. ADW: autoclaved distilled water; MRS: autoclaved MRS medium; ADW + Cr(VI): potassium dichromate in autoclaved distilled water (1 mM); MRS + TW1-1: TW1-1(1 × 10^9^ CFU/once every other day) in 0.25 mL MRS medium; MRS + LZU-D2: LZU-D2 (1 × 10^9^ CFU/once every other day) in 0.25 mL MRS medium. Mice received ADW (+Cr[VI]) *ad libitum*, and received MRS, MRS + TW1-1, and MRS + LZU-D2 via gavage.

Group	Treatment Groups (n = 10)	Drinking	Gavaging
1	Control	ADW	MRS
2	Cr(VI)only	ADW + Cr(VI)	MRS
3	Cr(VI) + TW1-1	ADW + Cr(VI)	MRS + TW1-1
4	Cr(VI) + LZU-D2	ADW + Cr(VI)	MRS + LZU-D2
5	TW1-1	ADW	MRS + TW1-1

### Determination of Cr(VI) concentrations in tissues and feces

Tissues and feces were harvested and stored in liquid nitrogen until use (Fig. S6). Frozen samples were thawed and air-dried in oven at 70 °C for 6 h until constant weight. The dessicated samples were then digested overnight in 10 ml of concentrated hydrochloric acid in enclosed caps. The digested solution was heated in a microwave until the solution was clear. The solution was then divided into two parts, one of which was treated with excess alkali, as Cr(III) can be precipitated to form insoluble oxides and hydroxides in water at neutral pH level^52^. The two parts were filtered through quantitative filter paper and diluted with distilled water to a volume of 25 ml, then analyzed using a graphite furnace atomic absorption spectrometer (ZEEnit®700 P Analytik Jena AG). The D-value between the two samples represented the Cr(VI) concentrations in tissues and feces.

### Estimation of enzyme levels and enzymatic activity in tissues

The activities of CAT, T-SOD, MDA, GSH and GSSG levels in the liver and kidneys were measured using commercial assay kits (Jiancheng Bioenginnering Institute, Nanjing, China). An enzyme-linked immunosorbent assay kit (Shanghai Enzyme-linked Biotechnology, China) was used to determine the TNF-α level in the liver, kidney, and small intestine. All experimental procedures were performed in strict accordance with the manufacturer’s instructions.

### Histological and TEM analysis

Histology of liver, kidney, and small intestine segments were carried out in standard procedures and sections were examined by light microscopy.

For TEM, tissues were immersed in 2.5% glutaraldehyde solution in PBS (Sigma, USA), and then embedded in epoxy resin and sliced with an ultramicrotome. TEM and energy-dispersive X-ray (EDX) spectroscopy analyses were performed by core facility of Lanzhou University.

### Quantification of TW1-1 population in feces

Total genomic DNA of cells in feces samples was extracted using a TIANamp Stool DNA kit (TIANGEN Biotech, Shanghai, China). 16S rRNA of TW1-1 was amplified by PCR and cloned to T vector (Takara, Dalian, China), and serially diluted and used as quantitative PCR templates to make a standard curve. qPCR was performed with SYBR Premix ExTa^TM^ II (TaKaRa, Dalian, China) on a real-time quantification PCR instrument (Bio-RAD CFX96, USA). The PCR program consisted of 95 °C for 30 s and 40 cycles at 95 °C for 5 s, 58 °C for 30 s, and 95 °C for 10 s. The copy numbers of the TW1-1 16S rRNA genes were calculated based on the standard curve and normalized against total 16S rRNA gene products amplified with universal 16 rRNA primers^53^. The primers are listed in Table 3.

**Table 3:** Primers.

DNA sample	Target	Primer	Sequence (5′—3′)	References
Fecal DNA	Total bacteria	Forward	GCAGGCCTAACACATGCAAGTC	50
		Reverse	CTGCTGCCTCCCGTAGGAGT	
Fecal DNA	TW1-1	Forward	GCATTAAGCATTCCGCCTGG	This study
		Reverse	ACCTGTATCCATGTCCCCGA	

### Illumina MiSeq sequencing of fecal bacterial 16S rRNA gene V4 region and data analysis

Genomic DNA was extracted and checked by PCR with universal 16S rRNA primers 27 F/1492 R. After confirmation, the DNA was lyophilized and sent for Illumina MiSeq sequencing (Nuozhou Biotech Co., Chengdu, China)^54,55^. Briefly, universal primers 515F-806R plus a 10-nt barcode were used amplify the hypervariable V4 region of the bacterial 16SrRNA gene. Each PCR was performed in duplicate, with the products recovered from the agarose gel, and then prepared for sequencing. The sequence data were processed using QIIME Pipeline v. 1.7.0 (http://qiime.org/). All sequence reads were trimmed and assigned to each sample based on their barcodes. Sequences of high quality (>150 bp in length, no ambiguous base “N,” and an average base quality score >30) were used for downstream analyses. Sequences were clustered into operational taxonomic units (OTUs) at a 97% identity threshold. The aligned ITS gene sequences were used for a chimera check with the Uchime algorithm. All samples were randomly resampled to 3,760 reads. Alpha-diversity measurements (Shannon), species richness (observed, chao1), and rarefaction curves were generated from the observed species. Taxonomy was assigned using the Ribosomal Database Project (RDP) classifier. Sequencing data of the bacterial communities was transformed to a quantitative matrix, and further analyzed by non-metric multidimensional scaling (NMDS), as NMDS analysis is generally considered to be the most effective ordination method for ecological-community data. The NMDS were conducted using the ‘vegan’ package (v. 2.0–10) in R software (v. 3.1.1).

### Statistical analysis

Data were expressed as the standard error of the mean (SEM) for each group. Analyses between groups were performed with one-way analysis of variance (ANOVA) tests using SPSS22. Differences were considered statistically significant at a P value < 0.05.

## Electronic supplementary material

Supplementary materials
