# Supplementary material for: Gut remediation: a potential approach to reducing chromium accumulation using Lactobacillus plantarum TW1-1
Source: Sci Rep. 2017 Nov 8;7:15000. doi: 10.1038/s41598-017-15216-9 (PMC5678100; doi:10.1038/s41598-017-15216-9)
Supplement: Supplementary file 1 — Supplementary materials [file 41598_2017_15216_MOESM1_ESM.doc]

**Gut remediation: a potential approach to reducing chromium accumulation using *Lactobacillus plantarum* TW1-1**

Gaofeng Wu ^1,2,+^, Xingpeng Xiao ^2,+^, Pengya Feng ^2^, Fuquan Xie ^2^, Zhengsheng Yu ^1^, Wenzhen Yuan ^3^, Pu Liu ^1,*^, Xiangkai Li ^1,*^

**SUPPLEMENTARY MATERIALS**

**Summary**

The supplementary information contains supplementary methods, five supplementary figures and three supplementary tables.

**Supplementary Methods**

**Determination of** **Cr(VI)-reducing abilities of TW1-1**

To determine whether the decrease in Cr(VI) concentrations was caused by biosorption or reduction, TW1-1 was cultured with 0.1 mM K_2_Cr_2_O_7_ for 48 h followed by centrifugation. The concentration of Cr(VI) in the supernatant was then measured, and the resulting cell pellets were lysed with 10 mg/L lysozyme (Takara, Dalian, China) at 37 °C for 1 h and then centrifuged. The concentration of Cr(VI) in the cell-lysate supernatant was immediately determined, following which the supernatant was oxidized by adding sodium hyposulfate (Zhiyuan Chemical Co., Tianjin, China). Concentrations of Cr(VI) were once again measured, which reflected the total amount of Cr in the cell lysate supernatant.

**Weight, drink, and food intake measurements and sampling**

Body weight, food and water intake, and stool appearance were documented for all mice every other day during the experiment. After 7 weeks, mice were placed in clean cages, and stool samples were collected and stored at –80°C. Mice were then sacrificed by cervical dislocation. The liver, kidney, and small intestine (jejunum) were collected from each mouse and divided into triplicate samples, with one stored in liquid nitrogen, a second stored in 2.5% glutaraldehyde, and the third fixed in 4% (w/v) paraformaldehyde at 4°C for later histological analysis. Sampling details are shown in Figure S1.

**Food and water intake and animal health conditions**

Rates of mortality of mice in the Cr(VI)-treated group were 20%, whereas no deaths occurred in the TW1-1-treated groups and the control group. Mice in the Cr(VI)-treated groups showed signs of constipation, and only small amounts of feces could be collected. Exposure to Cr(VI) and/or TW1-1 did not affect overall food/water intake as compared to that in mice in the control group (Figure S3). Moreover, little change in average body weight was observed (Figure S3).

**Supplementary Figures**


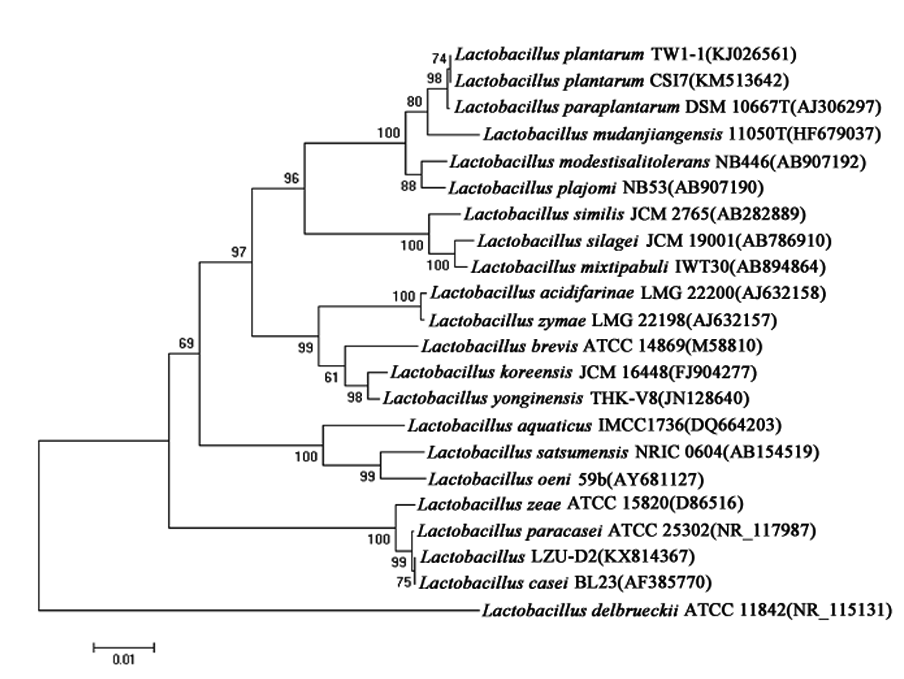


**Supplementary Figure 1.** Phylogenetic analysis of the selected strains based on the neighbor-joining method


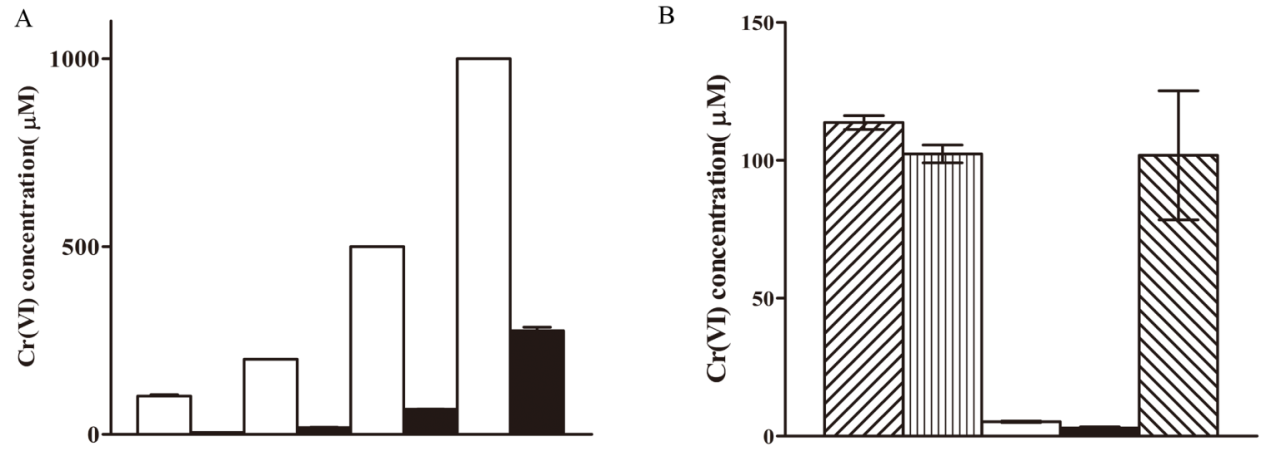


**Supplementary Figure 2.** The Cr reduction ability of *Lactobacillus plantarum* TW 1-l. A: The reduction ability of *Lactobacillus plantarum* TW 1-l under different Cr(VI) concentrations (100 μM, 200 μM, 500 μM, and 1000 μM). (□) Initial Cr(VI) concentration; (■) Cr(VI) concentration after incubation for 48 h. B: The Cr reduction ability of TW1-1 under 100 μM Cr(VI). (▨) Initial Cr(VI) concentration; (▥) Cr(VI) concentration after incubation for 48 h without TW1-1; (□) Cr(VI) concentration after incubation for 48 h; (■) Cr(VI) concentration absorbed by TW1-1; (▧) concentration of Cr(III). The average of three replicates and the standard deviation are presented.


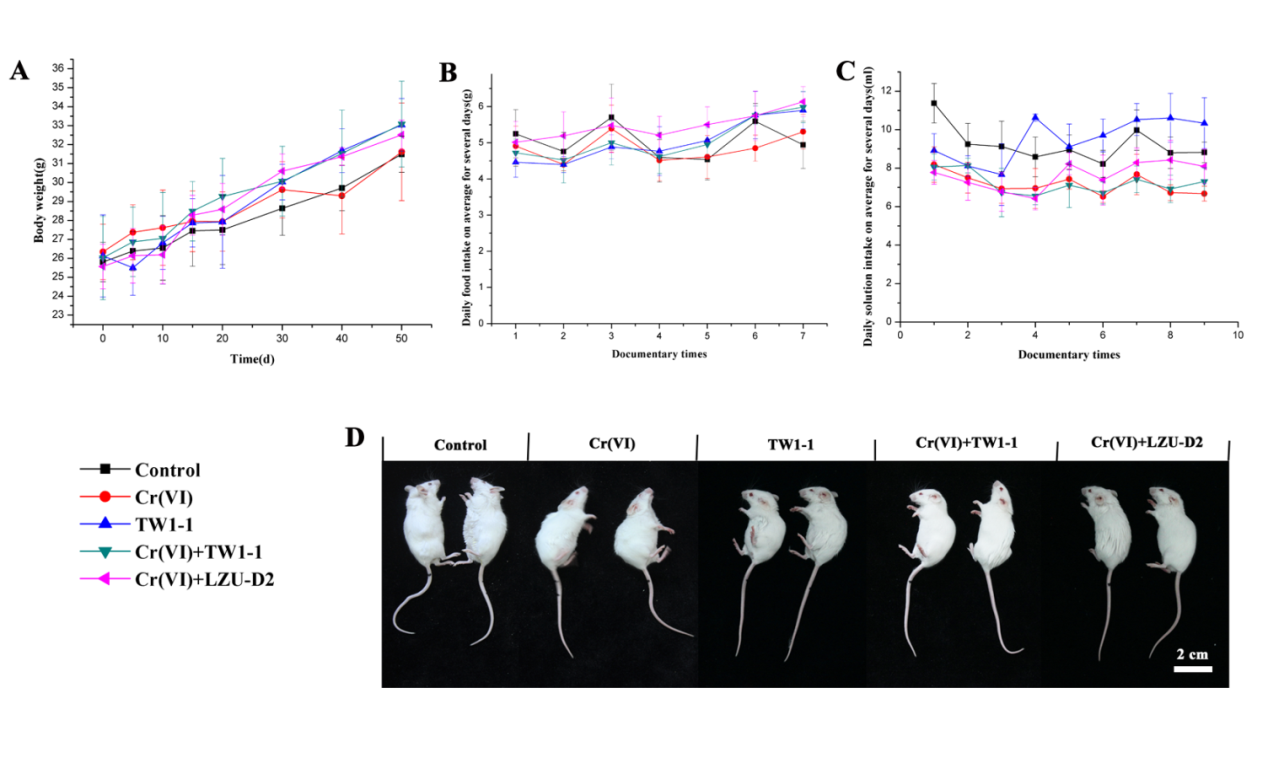


**Supplementary Figure 3** Food and water intake, and mice growth conditions. Photos were taken a week before mice were sacrificed.


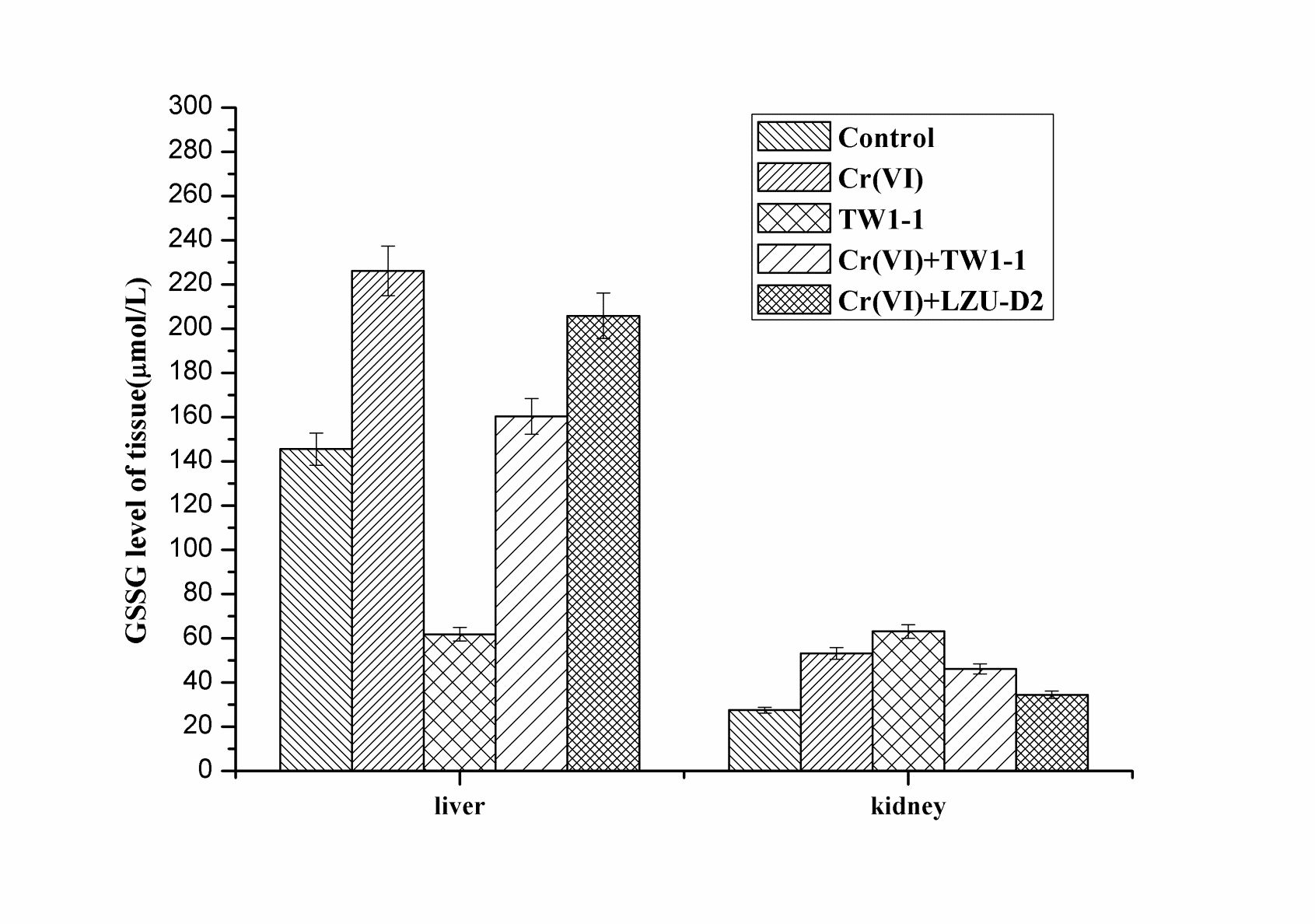


**Supplementary Figure 4** Effects of TW1-1 on Cr-induced alterations of the activities

of GSSG level in the liver and kidneys of mice.


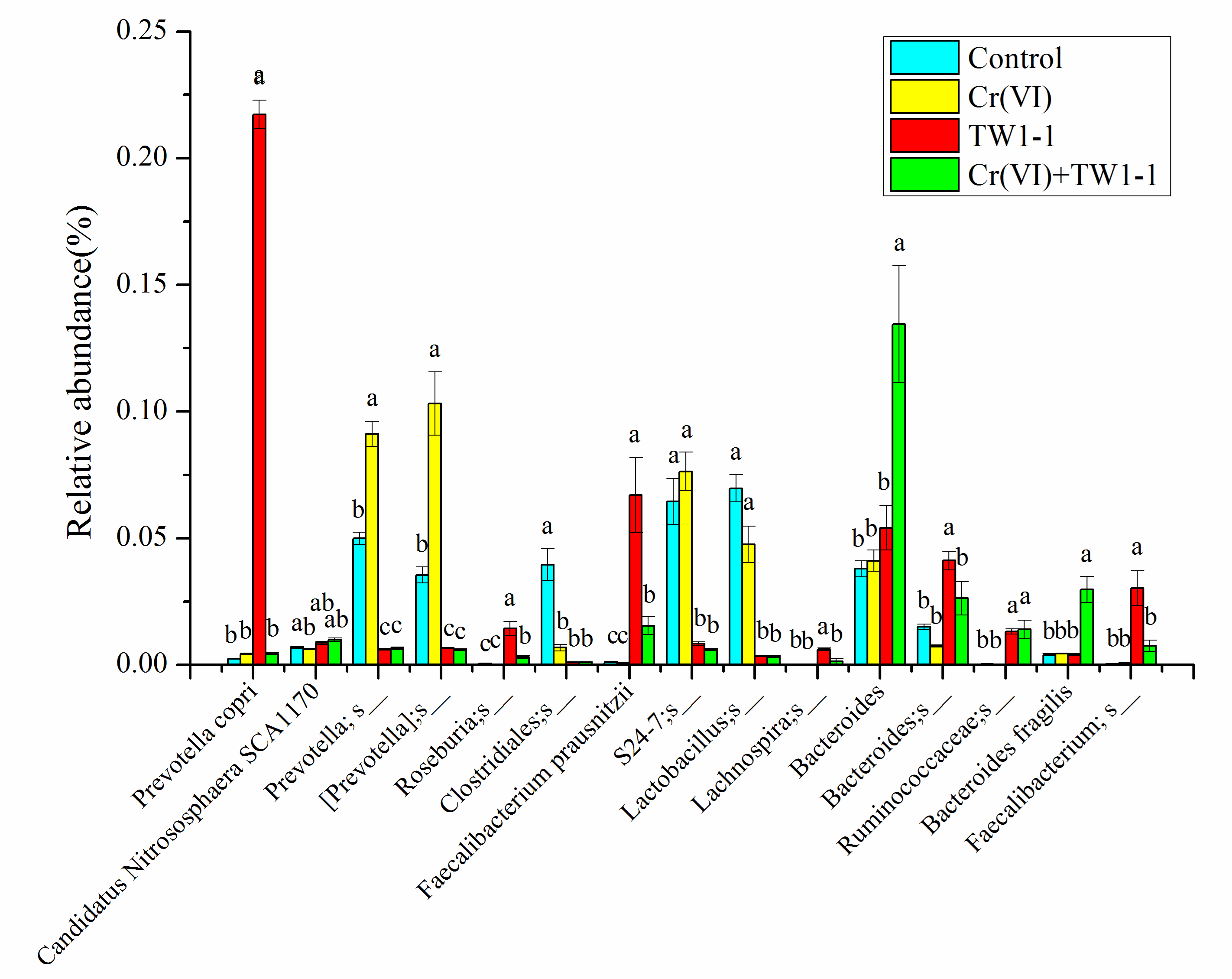


**Supplementary Figure 5** Relative abundance of bacterial 16S rRNA gene at the species level, showing the relative abundance changed by Cr(VI) or TW1-1. Same letters indicate there were no significant differences (P>0.05); different letters indicate a significant difference (P<0.05).


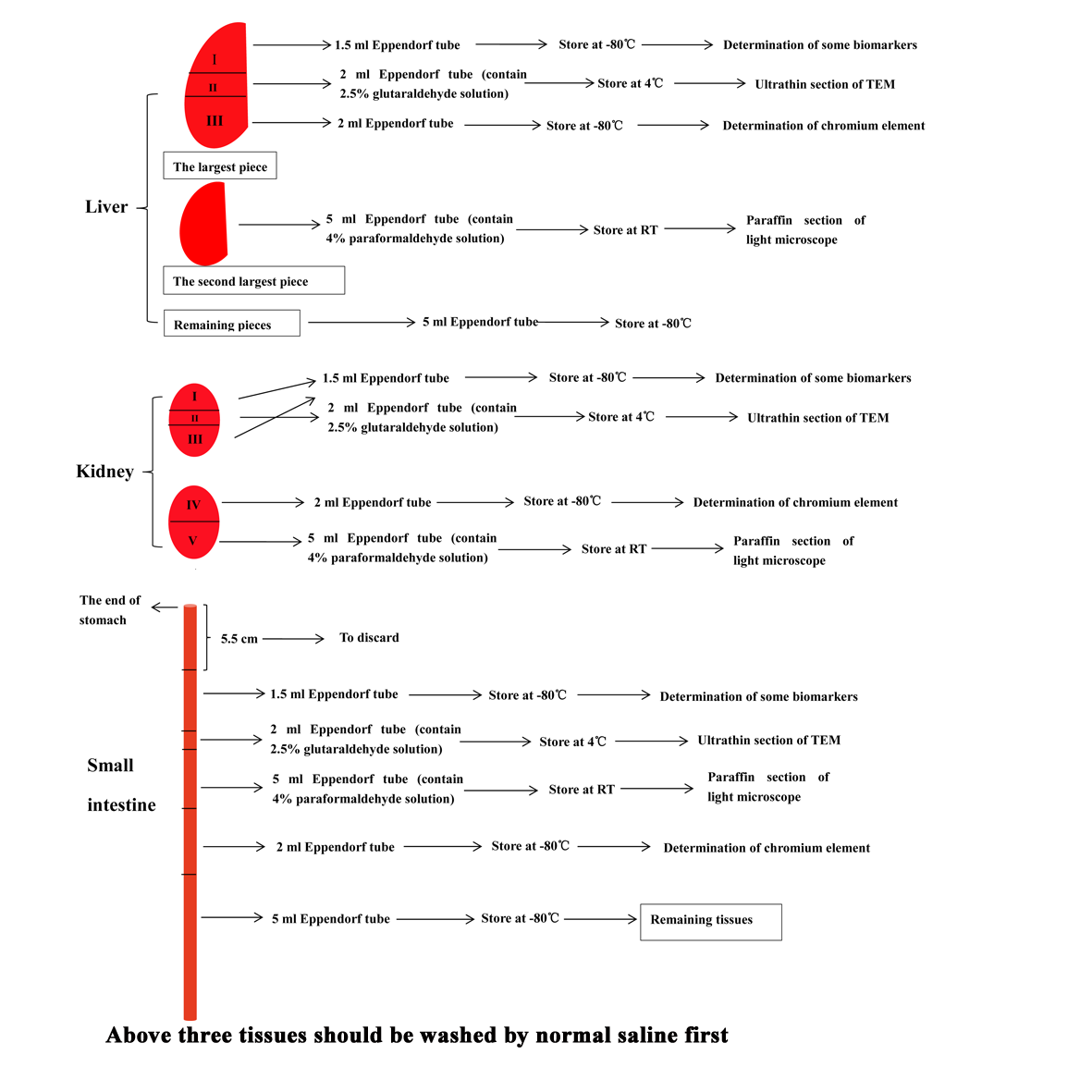


**Supplementary Figure 6.** Sampling details.

**Supplementary Tables**

**Supplementary Table 1** Chao1 and Shannon index for each sample

| Sample | Chao1 | Observed OUT | Shannon index |
| --- | --- | --- | --- |
| Control | 2007.53 | 864.1 | 8.34 |
| Control | 2091.06 | 935.5 | 8.83 |
| Control | 2246.15 | 1027.7 | 8.91 |
| Control | 2169.61 | 1011.3 | 8.8 |
| Control | 2304.6 | 940.7 | 8.47 |
| Cr(VI) | 2156.96 | 952.6 | 8.5 |
| Cr(VI) | 2212.73 | 874 | 8.31 |
| Cr(VI) | 2447.73 | 971.6 | 8.63 |
| Cr(VI) | 2349.95 | 960.1 | 8.55 |
| Cr(VI) | 2698.31 | 980.2 | 8.57 |
| TW1-1 | 2642.54 | 953.9 | 8.41 |
| TW1-1 | 2351.18 | 959.6 | 8.36 |
| TW1-1 | 2300.19 | 829.2 | 7.98 |
| TW1-1 | 2315.47 | 748 | 7.19 |
| TW1-1 | 1838.58 | 769.6 | 7.76 |
| Cr(VI)+TW1-1 | 2098.34 | 820.9 | 7.52 |
| Cr(VI)+TW1-1 | 2544.16 | 987 | 8.55 |
| Cr(VI)+TW1-1 | 1811.75 | 770.1 | 7.19 |
| Cr(VI)+TW1-1 | 2060.47 | 696.9 | 7.15 |
| Cr(VI)+TW1-1 | 2241.3 | 789.8 | 7.76 |

**Supplementary Table 2**

Relative abundances of gut microbial community at the family level.

| Taxon | Control | Cr(VI) | TW1-1 | Cr(VI)+TW1-1 |
| --- | --- | --- | --- | --- |
| *Cenarchaeaceae* | 0.0579±0.0218 | 0.0886±0.0185 | 0.0961±0.0296 | 0.05732±0.0234 |
| *Nitrososphaeraceae* | 1.5588±0.1907 | 1.454±0.0822 | 1.8294±0.1776 | 1.844±0.2219 |
| mb2424 | 0.0962±0.0104 | 0.0505±0.016 | 0.0896±0.0281 | 0.1088±0.0368 |
| Ellin6075 | 0.1087±0.0191 | 0.0698±0.0187 | 0.0833±0.022 | 0.0825±0.0214 |
| *Microbacteriaceae* | 0.0387±0.0122 | 0.0317±0.0101 | 0.0388±0.0125 | 0.0505±0.0124 |
| *Gaiellaceae* | 0.3021±0.0486 | 0.2024±0.041 | 0.2544±0.045 | 0.2464±0.031 |
| *Bacteroidaceae* | 7.8689±1.2358^c^ | 7.5534±1.052^c^ | 18.4023±1.6541^b^ | 42.6089±1.8735^a^ |
| *Porphyromonadaceae* | 0.7224±0.1422 | 1.4839±0.2275 | 0.7748±0.1757 | 2.055±1.0983 |
| *Prevotellaceae* | 6.9399±0.8488^b^ | 11.3209±1.0634^b^ | 28.6154±3.5578^a^ | 1.5747±0.0953^c^ |
| *Rikenellaceae* | 2.7691±0.4982^a^ | 3.5484±0.4584^a^ | 0.9538±0.1432^b^ | 0.4604±0.0634^b^ |
| S24-7 | 21.4135±2.5402^b^ | 30.703±2.264^a^ | 4.5366±1.0274^c^ | 2.3498±0.2462^c^ |
| *[Odoribacteraceae]* | 3.0939±0.5289^a^ | 0.5693±0.1132^b^ | 0.2463±0.0666^b^ | 0.377±0.1112^b^ |
| *[Paraprevotellaceae]* | 4.5306±0.7963^b^ | 10.506±1.1834^a^ | 1.1585±0.1521^c^ | 0.946±0.0792^c^ |
| *Cytophagaceae* | 0.7677±0.044^c^ | 0.9416±0.0809^b^ | 1.2623±0.0504^a^ | 0.9711±0.0573^b^ |
| *Flavobacteriaceae* | 0.4621±0.0494 | 0.6707±0.0473 | 0.5177±0.1054 | 0.5753±0.0754 |
| *Chitinophagaceae* | 0.6024±0.0438^b^ | 0.8541±0.0721^a^ | 0.754±0.0252^a^ | 0.7554±0.0357^a^ |
| *Saprospiraceae* | 0.0643±0.0269 | 0.0889±0.0236 | 0.0825±0.0291 | 0.0763±0.0129 |
| *Ignavibacteriaceae* | 0.1069±0.0066 | 0.116±0.0073 | 0.114±0.0186 | 0.0916±0.0139 |
| *Caldilineaceae* | 0.0577±0.0158 | 0.0758±0.0126 | 0.0646±0.0232 | 0.0703±0.0164 |
| A4b | 0.102±0.0181 | 0.0824±0.0276 | 0.0891±0.0127 | 0.0884±0.0302 |
| *Synechococcaceae* | 0.0582±0.024 | 0.0509±0.0163 | 0.0511±0.0225 | 0.0568±0.0181 |
| *Bacillaceae* | 0.2628±0.0279 | 0.2724±0.0377 | 0.425±0.0358 | 0.3118±0.089 |
| *Planococcaceae* | 0.1353±0.02887 | 0.095±0.0143 | 0.1027±0.0416 | 0.0957±0.0272 |
| *Lactobacillaceae* | 8.5193±1.3937^a^ | 2.6894±0.5796^b^ | 0.6595±0.1092^b^ | 0.6404±0.0703^b^ |
| *Leuconostocaceae* | 0.1536±0.0327 | 0.1139±0.0327 | 0.1611±0.0442 | 0.1585±0.0328 |
| *Streptococcaceae* | 0.2626±0.0521^a^ | 0.0714±0.0069^b^ | 0.3085±0.0771^a^ | 0.4207±0.0514^a^ |
| *Clostridiaceae* | 0.1092±0.0134 | 0.2862±0.1099 | 0.1718±0.0361 | 0.3453±0.2825 |
| *Lachnospiraceae* | 6.3407±0.8658^a^ | 2.2194±0.3241^b^ | 6.3433±0.6066^a^ | 6.5115±0.4279^a^ |
| *Peptostreptococcaceae* | 0.0574±0.0212 | 0.0759±0.0127 | 0.0638±0.0145 | 0.0948±0.0169 |
| *Ruminococcaceae* | 5.114±0.8371^b^ | 2.6403±0.3985^b^ | 18.7557±4.0071^a^ | 5.7508±0.9949^b^ |
| *Veillonellaceae* | 0.0566±0.0137^b^ | 0.0708±0.0129^b^ | 1.6174±0.5342^a^ | 2.6565±0.2993^a^ |
| *Erysipelotrichaceae* | 0.2244±0.0324 | 0.1517±0.0363 | 0.3707±0.1151 | 0.342±0.0535 |
| 0319-6A21 | 0.141±0.0376 | 0.1198±0.0377 | 0.162±0.0564 | 0.1467±0.0419 |
| *Nitrospiraceae* | 0.2115±0.0222 | 0.1895±0.0425 | 0.2375±0.0495 | 0.2727±0.0379 |
| *Pirellulaceae* | 0.0642±0.0118^b^ | 0.0953±0.0113^b^ | 0.1593±0.0265^a^ | 0.1193±0.0074^a^ |
| *Hyphomicrobiaceae* | 0.1866±0.0327 | 0.171±0.0331 | 0.1928±0.0526 | 0.2089±0.059 |
| *Rhodobacteraceae* | 0.0511±0.0076 | 0.0759±0.0127 | 0.0706±0.0281 | 0.0317±0.0099 |
| *Rhodospirillaceae* | 0.1475±0.0135 | 0.076±0.0079 | 0.1678±0.0474 | 0.1078±0.0235 |
| *Sphingomonadaceae* | 0.0895±0.006^b^ | 0.171±0.0262^ab^ | 0.1219±0.0287^ab^ | 0.2108±0.053^a^ |
| *Alcaligenaceae* | 0.185±0.0527 | 0.453±0.172 | 0.549±0.11 | 0.5783±0.2741 |
| *Comamonadaceae* | 0.2047±0.01175 | 0.2658±0.0412 | 0.2244±0.0558 | 0.2412±0.0169 |
| *Rhodocyclaceae* | 0.0645±0.0229 | 0.0443±0.0268 | 0.0383±0.0069 | 0.0379±0.0117 |
| *Syntrophobacteraceae* | 0.262±0.0374 | 0.2149±0.0268 | 0.3032±0.1075 | 0.2847±0.0445 |
| *[Entotheonellaceae]* | 0.0559±0.0069^b^ | 0.1693±0.0079^a^ | 0.1993±0.0108^a^ | 0.0639±0.0006^b^ |
| *Moraxellaceae* | 0.1596±0.0165 | 0.2154±0.0371 | 0.1651±0.0234 | 0.2542±0.0357 |
| *Pseudomonadaceae* | 0.0644±0.0178 | 0.0634±0.0201 | 0.1757±0.0439 | 0.1587±0.0468 |
| *Piscirickettsiaceae* | 0.09±0.0219 | 0.0947±0.0222 | 0.0447±0.0166 | 0.0759±0.0255 |
| *Sinobacteraceae* | 0.1927±0.0494 | 0.2216±0.015 | 0.2093±0.0459 | 0.2612±0.0521 |
| *Xanthomonadaceae* | 0.2381±0.0467 | 0.2775±0.0547 | 0.2469±0.0195 | 0.2348±0.0277 |
| *Mycoplasmataceae* | 0.3898±0.0779^a^ | 0.4414±0.053^a^ | 0.032±0.0005^b^ | 0.0534±0.0078^b^ |
| Unclassfied,others | 27.973±3.2307^a^ | 16.2884±0.8841^b^ | 10.3±1.1613^c^ | 10.663±1.2444^bc^ |

Same letters indicate no significant differences (*P>0.05*); different letters indicate a significant difference (*P<0.05*).

**Supplementary Table 3**

Relative abundance of gut microbes at the genus level.

| Taxon | Control | Cr(VI) | TW1-1 | Cr(VI)+TW1-1 |
| --- | --- | --- | --- | --- |
| *Nitrosopumilus* | 0.0579±0.0218 | 0.0886±0.0185 | 0.0899±0.0287 | 0.051±0.0193 |
| *Candidatus Nitrososphaera* | 1.5588±0.1907 | 1.4477±0.0829 | 1.8294±0.1776 | 1.844±0.2219 |
| *Bacteroides* | 7.8689±1.2358^c^ | 7.5534±1.052^c^ | 18.4023±1.6541^a^ | 28.0132±9.0885^b^ |
| *Parabacteroides* | 0.7031±0.1457 | 1.4586±0.2164 | 0.7563±0.172 | 2.0299±1.1081 |
| *Prevotella* | 5.7356±1.4138^b^ | 11.2829±1.0518^b^ | 28.6154±3.5578^a^ | 1.5747±0.0953^c^ |
| AF12 | 0.6679±0.2418^a^ | 0.3293±0.0915^a^ | 0.0322±0.0104^b^ | 0.038±0.0117^b^ |
| *Odoribacter* | 3.0939±0.5289^a^ | 0.5693±0.1132^b^ | 0.2761±0.0563^b^ | 0.2414±0.048^b^ |
| *Paraprevotella* | 0.1824±0.0703^ab^ | 0.3652±0.0332^a^ | 0.3349±0.0521^a^ | 0.1069±0.0086^b^ |
| *[Prevotella]* | 3.7943±0.6625^b^ | 809264±0.9816^a^ | 0.8034±0.07^c^ | 0.8029±0.0606^c^ |
| *Adhaeribacter* | 0.0577±0.0066 | 0.0569±0.0062 | 0.063±0.033 | 0.0573±0.0187 |
| *Flavobacterium* | 0.3339±0.0447^b^ | 0.5316±0.0558^a^ | 0.3578±0.0717^ab^ | 0.3863±0.0492^ab^ |
| *Synechococcus* | 0.0452±0.0167 | 0.0317±0.01 | 0.0384±0.0164 | 0.0505±0.0187 |
| *Bacillus* | 0.2246±0.0296 | 0.2407±0.0318 | 0.3617±0.0409 | 0.2793±0.0843 |
| *Lactobacillus* | 5.6404±2.0131 | 3.6124±1.5577 | 3.4579±2.4429 | 0.6278±0.069 |
| *Lactococcus* | 0.0573±0.027 | 0.0819±0.0288 | 0.091±0.0411 | 0.0951±0.0297 |
| *Clostridiaceae Clostridium* | 0.0449±0.0242 | 0.0633±0.0142 | 0.1083±0.0239 | 0.3073±0.2758 |
| *Lachnospiraceae Clostridium* | 0.1604±0.0234^b^ | 0.1835±0.0405^b^ | 0.2278±0.022^b^ | 1.3347±0.6318^a^ |
| *Coprococcus* | 0.4354±0.0738^ab^ | 0.2024±0.0754^b^ | 0.6513±0.1767^a^ | 0.2401±0.1759^ab^ |
| *Roseburia* | 0.1148±0.0293^c^ | 0.076±0.0239^c^ | 3.7498±0.5174^a^ | 1.3162±0.0383^b^ |
| *Faecalibacterium* | 0.1609±0.0378^b^ | 0.1514±0.0349^b^ | 10.8581±4.6576^a^ | 0.882±0.0355^b^ |
| *Oscillospira* | 3.577±0.5989^a^ | 1.4605±0.2255^b^ | 0.4304±0.0642^b^ | 1.6135±0.2601^b^ |
| *Ruminococcus* | 0.3076±0.0785 | 0.0817±0.0322 | 0.6125±0.3112 | 0.4137±0.1238 |
| *Phascolarctobacterium* | 0.0319±0.0001^b^ | 0.0378±0.0154^b^ | 0.4972±0.3034^a^ | 1.1583±0.3878^a^ |
| *Clostridium* | 0.1277±0.0221 | 0.0316±0.01 | 0.058±0.0283 | 0.3351±0.1942 |
| *Nitrospira* | 0.1793±0.0129 | 0.1517±0.0354 | 0.1799±0.0397 | 0.216±0.0278 |
| *Rhodoplanes* | 0.0772±0.0246 | 0.095±0.0176 | 0.0901±0.0316 | 0.1204±0.0364 |
| *Sphingomonas* | 0.0702±0.0117 | 0.1455±0.0162 | 0.0832±0.0261 | 0.1605±0.0619 |
| *Sutterella* | 0.1786±0.0566 | 0.453±0.172 | 0.5033±0.1263 | 0.572±0.2763 |
| *Acinetobacter* | 0.1149±0.0157^b^ | 0.1902±0.0338^ab^ | 0.1454±0.0269^ab^ | 0.2225±0.0419^a^ |
| *Mycoplasma* | 0.3247±0.0953^a^ | 0.4414±0.053^a^ | 0.0256±0.0064^b^ | 0.0534±0.0078^b^ |
| Unclassified,others | 57.0996±3.085^a^ | 51.065±2.8791^a^ | 26.6306±2.8072^b^ | 24.9325±2.8183^b^ |

Same letters indicate no significant differences (*P>0.05*); different letters indicate a significant difference (*P<0.05*).
